# Development of an Infrastructure for the Prediction of Biological Endpoints in Industrial Environments. Lessons Learned at the eTOX Project

**DOI:** 10.3389/fphar.2018.01147

**Published:** 2018-10-11

**Authors:** Manuel Pastor, Jordi Quintana, Ferran Sanz

**Affiliations:** Research Programme on Biomedical Informatics (GRIB), Institut Hospital del Mar d’Investigacions Mèdiques (IMIM), Department of Experimental and Health Sciences, Universitat Pompeu Fabra, Barcelona, Spain

**Keywords:** *in silico* toxicology, computational toxicology, predictive models, chemical safety, drug safety, industrial environments, public-private partnership, machine learning

## Abstract

*In silico* methods are increasingly being used for assessing the chemical safety of substances, as a part of integrated approaches involving *in vitro* and *in vivo* experiments. A paradigmatic example of these strategies is the eTOX project http://www.etoxproject.eu, funded by the European Innovative Medicines Initiative (IMI), which aimed at producing high quality predictions of *in vivo* toxicity of drug candidates and resulted in generating about 200 models for diverse endpoints of toxicological interest. In an industry-oriented project like eTOX, apart from the predictive quality, the models need to meet other quality parameters related to the procedures for their generation and their intended use. For example, when the models are used for predicting the properties of drug candidates, the prediction system must guarantee the complete confidentiality of the compound structures. The interface of the system must be designed to provide non-expert users all the information required to choose the models and appropriately interpret the results. Moreover, procedures like installation, maintenance, documentation, validation and versioning, which are common in software development, must be also implemented for the models and for the prediction platform in which they are implemented. In this article we describe our experience in the eTOX project and the lessons learned after 7 years of close collaboration between industrial and academic partners. We believe that some of the solutions found and the tools developed could be useful for supporting similar initiatives in the future.

## Introduction

*In silico* methods are increasingly being used in the assessment of the chemical safety of chemicals as a part of integrated approaches, in which computational tools are used to synergically complement the experimental methods, with the aim of generating better and more efficient predictions of the potential toxicological liabilities of the compounds under study (Luechtefeld et al., 2018). Recent advances in machine learning and deep learning methodologies are demonstrating their effectiveness in this respect (Lenselink et al., 2017; Liu et al., 2017). Moreover, ambitious collaborative initiatives in this field have been set up with the aim of increasing the availability of relevant data frameworks and developing the aforementioned integrative approaches on top of those data. Among these initiatives, EU-ToxRisk (Daneshian et al., 2016), HESS (Sakuratani et al., 2013), TransQST (Maldonado et al., 2017), iPiE (Bravo et al., 2016) and eTOX (Cases et al., 2014; Sanz et al., 2017; Steger-Hartmann and Pognan, 2018) deserve to be highlighted.

In particular, the eTOX project, funded by the Innovative Medicines Initiative, constituted a pioneering exercise of extracting and integrating *in vivo* data from legacy resources at the pharmaceutical industry, and exploiting such data for read-across and the development of predictive models, since one of the aims of the eTOX project was to set up an integrated system for the prediction of toxicological endpoints, with a focus on organ and *in vivo* endpoints. The project faced many challenges, some of which have been described in previous publications (Cases et al., 2014; Sanz et al., 2015, 2017; Steger-Hartmann and Pognan, 2018). Here we wish to share our experiences in an aspect that is often overlooked in this kind of projects, which is how to translate predictive models generated by academia or by Small-Medium Enterprises (SMEs) into a production environment where they can be routinely applied. Irrespectively of the scientific quality of a model, it must meet several requirements to make it amenable for being used by the pharmaceutical industry. This requires building a common understanding between academic and industrial partners, identifying the end-user needs, and making significant efforts to incorporate into the models and the predictive system features that, in spite of their low scientific interest, make the difference between usable and not usable models. In the present article we describe the most significant lessons learned in eTOX, describing some of the problems we identified and describing the solutions applied to solve or mitigate them. Most of these solutions are the result of long hours of discussion, where we learned to understand each other’s points of view.

## Results

Developing a computational model for predicting a biological endpoint is a complex task. In the case of QSAR-like models, their development involves (at least) the curation of the training series, the selection of appropriate molecular descriptors and machine learning methods, building, validation, and interpretation of the model. However, when the aim is to produce a model that can be used by people outside of the modeler’s laboratory, the work has not finished with the generation of the model. There is an additional difficulty if we intend to use the models in industrial environments, particularly if the structures of the compounds should be treated as confidential.

In the following sections we will discuss issues related with the model development and implementation, the need of a standard modeling framework for supporting model development and maintenance, as well as the model documentation and validation. In the last section we will discuss also the problems related with the confidentiality of the structures for model training and application.

### Platform for Model Development and Production

Most of the eTOX models were developed by academic partners and SMEs, located in different European countries. Therefore, the architecture of the system to be developed should support independent and concurrent model development, while model prototypes should be made accessible to the end users (pharmaceutical companies) for early testing. This software platform, designed to increase the model development efficiency, should be compatible with the local deployment of the final system. The final version must be installed physically at the computational resources of the pharmaceutical companies, since the end users considered that only an installation behind the company firewalls guarantees that they could be used on highly confidential compounds corresponding to drug candidates under development.

**FIGURE 1:**
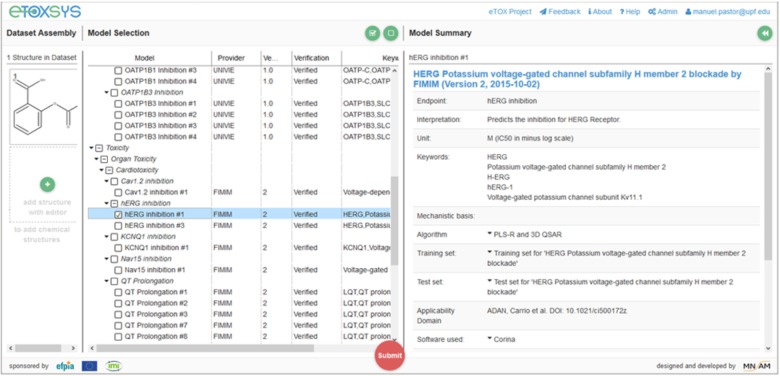
Graphic User Interface (GUI) of eTOXsys for using the eTOX models.

These requirements made necessary the adoption of technical solutions that facilitate the remote access to the models and the portability of its software implementation, which consisted of two layers of containerization. The outer layer consisted in a self-contained virtual machine (VM), configured to expose a REpresentational State Transfer (REST) web service (Fielding, 2000) to predict the properties of query compounds. VMs were installed at the partners facilities, thus making possible that models developed at remote sites were immediately accessible through a centralized web server which shows all available models through a single graphical interface (see **Figure 1**). The physical location of the server running the computations was completely transparent to the end user.

**FIGURE 2:**
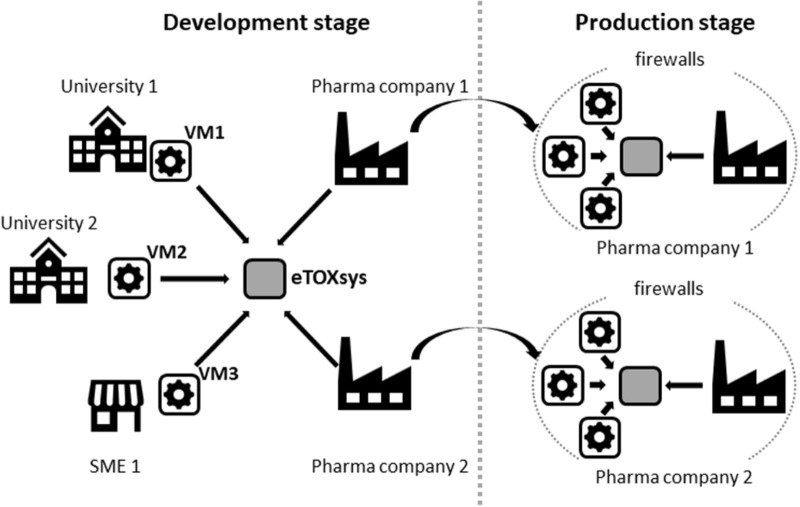
Scheme of the eTOX development and production setup.

**Figure 2** shows a schematic representation of the setup that was adopted for the development and production of the eTOX models.

The eTOX development setup has the inconvenience that it cannot guarantee an appropriate level of confidentiality on the query structures. These are sent over the Internet, and the computations are carried out in academic servers, some of which do not comply with the strict security requirements necessary to protect confidential structures. For this reason, the testing of the models was carried out using only non-confidential structures and the user interface shows a disclaimer informing of the security risks.

The final version of the system, as mentioned before, was installed locally at the computational facilities of the EFPIA partners (**Figure 2**). The deployment of the system was facilitated by the use of VMs, which can run in heterogeneous computational environments (i.e., diverse operative systems and hardware configurations). The VMs were relatively compact (between 4 and 5 Gb each) and did not have high computational needs (recommended settings were 1 CPU and 2 Gb RAM per VM). The whole system can be accommodated in low-end computational clusters or even in an isolated server with multiple CPUs.

**FIGURE 3:**
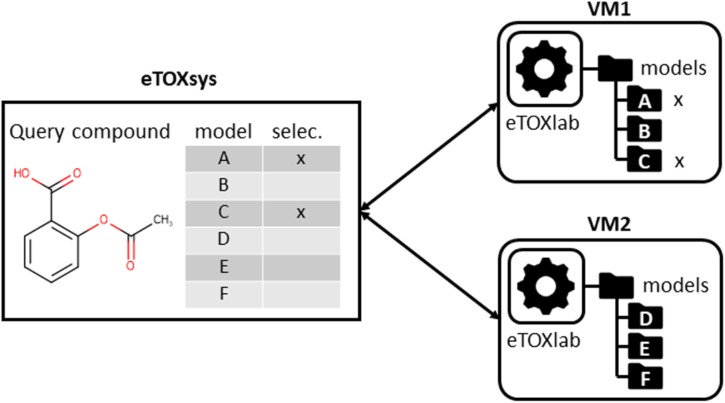
Scheme of eTOXlab location within the virtual machines (VMs), storing multiple models and serving them as web services.

In the same way that the VMs provided a layer of standardization for the external access to the models, we had the need of developing an *ad hoc* modeling framework, called eTOXlab (Carrió et al., 2015), which supports modelers in their task of implementing and maintaining the predictive models within the VMs. Essentially, each VM contains an instance of eTOXlab, which can manage multiple models and exposes them as web services using a standard Application Programming Interface (API), as shown in **Figure 3**. All the model inputs and outputs are redirected trough the web services. Therefore, as far as the models are correctly implemented within eTOXlab, they are perfectly integrated into the project predictive platform and visible in the common interface shown in **Figure 1**.

### Model Development and Maintenance

**FIGURE 4:**
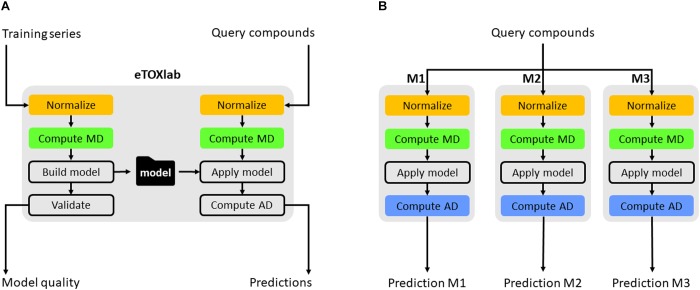
Scheme of eTOXlab workflow **(A)**. Query compounds can be submitted to multiple models (M1–M3), obtaining multiple predictions **(B)**.

Apart from connecting the individual models to the eTOXsys prediction system, eTOXlab provides additional support for the model development, maintenance, and documentation. Regarding model development by diverse teams of modelers, it is important to make use of common tools providing consistent solutions for tasks that need to be carried out by the different models. An example of this is the structure normalization, since the end user expects that the input structure is internally normalized and processed in the same way by all the models to which it is submitted. The use of a common modeling framework allows employing a common workflow for the building of all the models and for carrying out predictions with them, where the same software tools are used at each step, thus guaranteeing that the results are consistent; an example is structure normalization. Classically, 2D structures of the molecules are entered by the end user using SMILES or SDFiles formats. Before these structures can be processed, they need to be submitted to a normalization protocol that takes care of removing counterions, saturating and ionizing the molecule to a certain pH and, in some cases, generating 3D structures. Ideally, query molecules must be submitted to the same protocol that was applied to the structures of the training series used for developing the models. When the same query molecule is submitted to multiple models at the same time, the protocols must also be consistent. This requirement is easily met by using the eTOXlab modeling framework. Models implemented in eTOXlab make use of a consistent workflow (**Figure 4**), which processes input molecules in sequential order, submitting them to a normalization tool, an ionization tool and a 3D conversion tool. The tools applied, and the precise parameters used can be customized for each model and are adequately documented, thus guaranteeing a fully consistent treatment in the model training and prediction.

The use of eTOXlab also allowed developing specific components for common tasks. An example of this is ADAN (Carrió et al., 2014), a method specifically developed for assessing the applicability domain of the predictive models developed in eTOX, which is able to generate robust reliability scorings for the predictions. In summary, the ADAN method is based on assessing how far is a query compound from the model applicability domain and, based on this, provide reliability indexes to the predictions. The reliability is translated to pseudo 95% Confidence Interval (CI), thus facilitating the appraisal of the prediction obtained. The ADAN methodology can also be applied to non-QSAR models (Capoferri et al., 2015).

**FIGURE 5:**
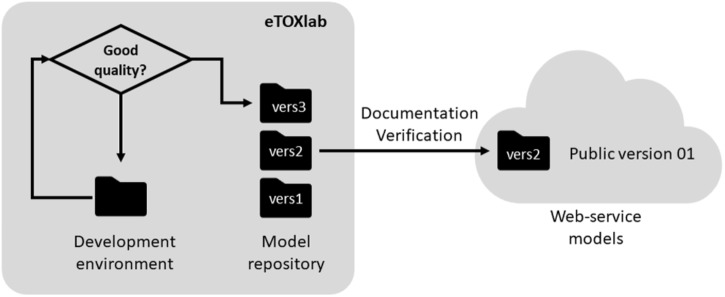
Life cycle of the eTOX models.

Another task that can be facilitated by the use of a modeling framework like eTOXlab is the maintenance of the models. Given that models are not static entities, once they are developed, they should evolve along the time by incorporating new compounds to the training series, updating of the software used at the different steps or refining the modeling workflow. In any case, every improvement produces a new version of the original model (see **Figure 5**).

In production environments, where important decisions can be based on model results, it is important to maintain a well-ordered inventory of all models and versions developed and use unique identifiers for each of them. As a minimum, the system must allow to reproduce predictions made by any model version.

In eTOX, every model was documented in a central repository, called eTOXvault, where it was assigned a unique public identifier and version number. For models developed within eTOXlab, two circuits of versioning were used. When a model was in development, all the files were stored in a specific development environment (so called “sandbox”). Only models that meet certain quality criteria were copied into a permanent storage space and assigned an internal, sequential version number. Initially these identifiers and version numbers were internal, as they were not exposed to anybody except to the model developer. Once the models were properly documented and verified (as described below), they were assigned an official identifier and version number and they were published as a web service visible to all consortium partners.

### Model Documentation and Validation

It is widely accepted that models must be documented. However, we learned that different actors have different expectations and very diverse needs regarding model documentation. Most end users require simple documentation describing, in a concise and non-technical language, what is the precise meaning of the model predictions and how reliable are those. On the other hand, modelers need to document the models at a more detailed level to allow reproducing the models and to facilitate the model maintenance. Potential future uses of the model results for regulatory purposes, recommend following widely recognized standards, such as the Organization for Economic Co-operation and Development (OECD) guidance document about QSAR modeling (Organisation for Economic Co-operation and Development, 2007), the guidance on the development, evaluation, and application of environmental models published by the US Environmental Protection Agency (EPA) (Environmental Protection Agency, 2009), or the requirements of the European REACH (Benfenati et al., 2011), or the recent efforts from the pharmaceutical industry (Myatt et al., 2018). In eTOX, models were documented following the OECD guidelines, but the sections of the document were reorganized in a way that allow to obtain summary extractions, as we described in a previous paper (Sanz et al., 2015).

To validate a model means to determine if the model is “fit-for-purpose.” This task is highly dependent of the use context and cannot be carried out in a general manner for all models. In eTOX the model validation was replaced by a systematic model verification methodology, which guarantees that the model produces the results described in the documentation (Hewitt et al., 2015).

### Structure Confidentiality

The eTOX project was a collaborative effort involving several major pharmaceutical companies, which contributed data generated and stored in-house for the training of predictive models. Sharing this information posed a major problem, in particular when it involved the structure of confidential compounds. Predictive models should ideally be built using all available structures and biological annotations available, irrespectively of the partner who contributed this information. Unfortunately, the data protection policies of the different industrial partners imposed obvious limitations, difficult to overcome.

At the beginning of the project we hoped to be able to develop and implement new structure-masking algorithms able to hash the structures into representations usable for building models, but resilient to any effort to reverse-engineer the algorithm and guess the original chemical structure. Our hope was not unfounded, and different similar methods have been published in the past (Tetko et al., 2005; Masek et al., 2008). For this particular purpose, we obtained excellent results using a simple random permutation of the molecular descriptors generated by methods like GRIND or GRIND2 (Pastor et al., 2000; Durán et al., 2008). The permuted vector of descriptors does not allow guessing the original structure, since the permutation destroyed any link between the value of the variables and their physico-chemical interpretation. Moreover, this approach is resistant to brute-force methods (Faulon et al., 2005; Filimonov and Poroikov, 2005), since these methods require the application of the same algorithm to a comprehensive database of structures, and a key element of the hashing algorithm (the random seed of the permutation) is never shared or revealed. The robustness of the algorithm was carefully tested and further demonstrated by code-breaking challenges at the project consortium level, where the hashed representation resisted any effort to identify the original structure. In these exercises, we also demonstrated that the hashed representation preserved all the information existing in the original molecular descriptors, and the models derived from them had equivalent quality.

Unfortunately, in these exercises we found that, beyond the robustness of our masking algorithm, it was impossible to convince the pharmaceutical companies to implement it in the eTOX project since, given the high corporate sensitiveness on the issue, such implementation would require costly external audits that we could not afford. For this reason, we adopted an alternative approach: if the confidential data cannot be taken out of the companies’ internal repositories, we can move the whole model building system to the companies, so the models can be built there. Indeed, we took advantage that the eTOXlab-VM containers are already portable model building engines. Without any modification, they can be used to develop fully functional models behind the companies’ firewalls. Furthermore, this approach could be even better if the models obtained could be shared without compromising the confidentiality of the training series. In order to make this possible, eTOXlab implements a “safe mode” for building models in a special way, which retains no information at all about the structures or identities of the training series. When configured in this way, the eTOXlab model consists in a small text file, with the values of the coefficients that must be applied to the molecular descriptors computed for future query compounds to estimate their biological activity. This small file can be exported to other partners without any risk since it is easy to audit to guarantee that no sensitive information at all is exported even using an unsecure means (e.g., e-mail, portable USB device).

## Discussion

Some of the solutions applied in eTOX for generating a predictive system usable in production environments involve the use of specific software, wrapping the scientific work developed by SMEs and academic partners into a “package” easier to deploy and integrate in corporate settings. The use of this kind of software, which is described in this article as a “modeling framework,” adds further advantages, like facilitating the consortium-wide adoption of standard modeling components, and simplifying key steps of the model life cycle, like the model retraining and maintenance. In eTOX, a new modeling framework was developed *ad hoc* for the project (eTOXlab). This software has been released as open source under GNU GPL v3.0 (GNU GPL v3, 2007). The source code of eTOXlab is accessible at https://github.com/phi-grib/eTOXlab. A fully configured VM including eTOXlab is also accessible at http://phi.upf.edu/envoy/. Hence, future projects aiming to develop similar predictive systems have now the option of reusing these resources, either as they are or customizing them to meet specific project needs.

We consider that these resources have value on their own, but they have an additional value as a proof-of-concept, since they demonstrate that they are helpful for making software tools developed by academic and SMEs usable by pharmaceutical companies. **Table 1** lists some of the key features that, in our opinion, such kind of frameworks must incorporate.

**Table 1:** Features required for the building of a predictive system usable in production environments.

Predictive system component	Feature	Importance
Framework	Support for model development at the academic/SMEs	Must
	Support for model deployment at the end-user site	Must
	Flexible enough to accommodate all modeling methodologies	Must
	Easy model maintenance and retraining	Must
	Pluggable components	Optional
Protocols	Model documentation	Must
	Prediction uncertainty	Must
	Use of international standards (QMRF/QPRF)	Depends on intended use


Another key element required is the definition and consortium-wide adoption of protocols for labeling, documenting and verifying the models. These are important aspects, which must be negotiated with the end-users for providing fit-for-purpose solutions. In this dialog, the expected use of the predictive system must be identified as soon as possible, since a modeling system aiming to prioritize lead compounds has completely different requirements, in terms of documentation and verification, than a system supporting decisions that could be communicated to regulatory agencies. This consideration should not be interpreted as a justification for considering optional the complete model documentation or the quantification of the prediction uncertainty; however, the standards used in either case are different. For this reason, the requirements derived from all intended model uses must be identified with the help of the end users, clearly defined and translated into system specifications.

One of the most complex aspects in the development of the aforementioned prediction systems is the internal adoption of the models by the end-users. The procedures vary from company to company, although they typically involve the validation of the system by comparing the prediction results with other *in silico* or experimental methods. As the structures being used in this comparison are often confidential, in the vast majority of cases the results of such validations are not made public. This is understandable but unfortunate, because this behavior results in a lack of feedback about the final usefulness of the predictive system. A published example of this kind of internal validations was the one carried out by Sanofi on the eTOX QT prolongation model using 434 drug candidates (Amberg et al., 2016).

Another aspect, briefly discussed in this article, is the potential use of portable modeling environments for building and sharing predictive models in which confidential structures are used. In eTOX, this was considered the only acceptable option, while solutions attempting to obfuscate, mask or encrypt the structures (or the molecular descriptors) were considered by the partners too risky to be used in practice. eTOXlab was configured for producing shareable models, which can be safely shared and exported because they contain no trace of the original structures. Similar features can also be easily implemented in other modeling frameworks. Here we want to emphasize the conceptual value of the aforementioned strategy consisting in building the models within the companies and exporting only the model coefficients. The implementation of this strategy only requires the use, across the collaborating partners, of a common modeling framework facilitating the import and export of the model coefficients.

Many of the eTOX partners have continued their collaboration and now participate in a new IMI project (eTRANSAFE)^1^, which shares with eTOX the aim to develop predictive systems. The ideas and principles described in this article are being applied, extending and adapting them to meet the objectives of this new project. One part of this effort is the development of a new modeling framework (called Flame), inspired on the same principles of eTOXlab but technologically more advanced. The source code of this software, still in development, is distributed under GNU GPL v3.0 (GNU GPL v3, 2007) and can be accessed at https://github.com/phi-grib/flame.

Finally, a limited version of eTOXsys, including the modeling system described here and a few selected models has been made open to the scientific community and can be accessed at http://etoxsys.eu/.

## Conclusion

Beyond the concrete database, predictive models and integrated computational system that have been developed, the eTOX project has demonstrated that the successful completion of ambitious industry-oriented collaborative projects requires not only the development and implementation of state-of-the-art scientific approaches, but also the careful implementation of adequate technical and organizational solutions. Among them, the adoption of adequate standards and protocols is a key component. The efforts done in eTOX in this respect are being extended to the new IMI eTRANSAFE project^1^, which will jointly exploit preclinical data and clinical safety information for a better prediction of potential human safety liabilities (Sanz et al., 2017).

We hope this paper will contribute to save the readers’ time and effort in similar public-private projects, as well as to improve the efficiency in the collaboration between the pharmaceutical industry and external parties in the development and application of computational tools supporting the drug discovery and development pipeline.

## Author Contributions

FS was the academic coordinator of the eTOX project. MP is a major contributor to the design of the eTOX predictive system described here, even if the credit belongs to the whole eTOX consortium. MP wrote, designed the figures and assembled this manuscript, which was enriched, refined and formatted by FS and JQ.

## Conflict of Interest Statement

The authors declare that the research was conducted in the absence of any commercial or financial relationships that could be construed as a potential conflict of interest.

## Footnotes

1 http://etransafe.eu
